# Predicting Risk for Reading Difficulty among Spanish-English Speaking Students: Classification Accuracy of Kindergarten Oral Language and Early Literacy

**DOI:** 10.1007/s40688-026-00592-9

**Published:** 2026-05-01

**Authors:** J. Marc Goodrich, Lisa Fitton, Nuria Gutiérrez, Erin Smolak, Gina Crosby-Quinatoa

**Affiliations:** 1 Department of Teaching, Learning, & Culture, Texas A&M University, 308 Harrington Tower, 4232 TAMU, College Station, TX 77843-4232 USA; 2 University of South Carolina, Columbia, USA; 3 Florida State University, Tallahassee, USA

**Keywords:** Bilingual, Oral language, Literacy, Screening, Reading difficulty

## Abstract

Assessment and identification of language and reading disabilities among young bilingual children represent a significant challenge. Research indicates that language and reading disabilities are frequently misidentified in this population (e.g., Mancilla-Martinez et al., 2023). Misidentification has serious consequences, as children with significant learning needs may not receive appropriate supports. Therefore, the purpose of this study was to identify which kindergarten early language and literacy measures have good classification accuracy for predicting first grade reading difficulty among Spanish-English bilingual children. Two hundred twenty children across South Carolina and Texas participated in this study. Children were enrolled in kindergarten, when they completed measures of oral language and early literacy skills in Spanish and English, and followed through first grade, when they completed measures of Spanish and English reading skills. Approximately 60% of the sample was enrolled in dual language instructional programs, and the remainder of the sample was enrolled in English-only programs. Results indicated that kindergarten English rapid letter naming had strong classification accuracy for distinguishing risk for dyslexia at the end of first grade among children enrolled in Spanish-English dual language instruction. However, no measures had strong classification accuracy for distinguishing children at risk for reading difficulty in English-only instructional contexts. These results have clear practical implications for screening bilingual students for risk for dyslexia and highlight the importance of considering language of instruction when evaluating risk for reading difficulty.

Reading ability ranges along a continuum (Elliott and Grigorenko, 2024), and involves several skills, with oral language skills and decoding skills serving as the foundation for reading comprehension (Hoover & Gough, 1990). The initial years of schooling are critical for establishing these foundational skills, and schools must make informed decisions about which students require additional instructional supports to prevent or remediate reading difficulties early in elementary school. For those students who are at the lower end of the reading distribution, early detection is crucial to ensure that they receive the necessary educational support to prevent them from falling behind (Catts et al., 2015; Lovett et al., 2017). However, given the finite nature of educational resources, schools must make decisions regarding the threshold ability for a student to receive a more personalized intervention. Typically, within the frameworks of Response to Intervention (RtI) and Multi-Tiered Systems of Support (MTSS), it is the lowest 20–25% of the student population that is targeted for additional support. If these students do not respond to this initial support, more individualized intervention is considered. These systems have proven to be effective for improving reading outcomes (Gersten et al., 2020), but an important first step for these systems to be effective is the accurate identification of those students at risk of developing reading difficulties.

Within RtI and MTSS frameworks, initial identification of risk for reading difficulty is accomplished via a process of universal screening, requiring a screening measure that is both efficient to administer to all the students in a classroom and accurate in classifying at-risk students. An important consideration in universal screening is the linguistic diversity present in U.S. classrooms. As of 2021, approximately 21% of U.S. school-age children speak a language other than English at home (Federal Interagency Forum on Child and Family Statistics, 2023). The majority (76%) of these students are Spanish speakers (National Center for Education Statistics, 2024). Notably, multilingual learners in U.S. schools experience heightened risk for both under-identification and over-identification in special education (Ortiz et al., 2011; Sullivan, 2011). Therefore, the purpose of this study was to evaluate the classification accuracy of various approaches to screening for reading difficulty among Spanish-English bilingual kindergarten students. Recognizing that many schools and communities include students exposed to multiple languages, this paper specifically examines bilingual kindergarten students for whom Spanish is the primary home language, attending school in the U.S. Throughout this paper, we will refer to this group of students as ‘bilingual.’

## Early Screening for Reading Difficulties: Classification Accuracy

A common approach to assessing the classification accuracy of a screening measure is ROC (Receiver Operating Characteristic) curve analysis (Johnson et al., 2009). This approach relies on a confusion matrix, which categorizes the classification results into True Positives (TP, students correctly identified as needing support), False Positives (FP, students incorrectly identified as needing support), True Negatives (TN, students correctly identified as not needing support), and False Negatives (FN, students in need of support who are not identified). There are several key metrics derived from this confusion matrix that allow researchers and practitioners to evaluate and optimize the classification accuracy of a screener, including sensitivity (the proportion of students needing support who are accurately identified, TP/[TP + FN]) and specificity (the proportion of students who do not need support who are accurately identified, TN/[TN + FP]). Additionally, the area under the ROC curve (AUC) provides an overall measure of a screening test’s diagnostic accuracy. AUC values approaching 1 suggest that the screening accurately classifies students at risk versus not at risk; in contrast, an AUC value of 0.50 implies that the screening’s predictive power is equivalent to random chance. Regarding the optimal value of these indicators, it is often recommended that early literacy screeners should achieve a sensitivity of at least 90%, a specificity of at least 80%, and an AUC larger than 0.85 (Compton et al., 2010; Jenkins et al., 2007). However, there is some disagreement in the literature (January & Klingbeil, 2020), likely stemming from the fact that these thresholds must remain flexible to accommodate variations in available resources and the unique demands of different community contexts. For example, some researchers have argued that lower specificity is acceptable in the context of response-to-intervention frameworks (Catts et al., 2009). In this situation, students who represent false positives (i.e., were identified by the screener as at risk but are not actually at risk) will be quickly identified when provided with Tier 2 instructional supports and can be moved back to Tier 1 with minimal loss of resources.

## Early Screening in a Bilingual Context: Challenges

Early screening of bilingual students poses significant challenges. Bilingual students are frequently overidentified with learning disabilities in the U.S., especially after third grade (National Center for Learning Disabilities, 2020; Rodríguez & Rodríguez, 2017; Samson & Lesaux, 2009; Sanatullova-Allison & Robison-Young, 2016). Paradoxically, this overidentification often stems from an initial underidentification from kindergarten through second grade, resulting in insufficient early attention to the needs of these multilingual learners (Richards-Tutor et al., 2013). Several systemic issues likely contribute to this early misidentification, including the variety of instructional programs and the lack of reliable assessments for this population. Additionally, the linguistic diversity within the multilingual population further challenges early identification. Bilingual learners often display different levels of proficiency in each language depending on the amount of exposure they have had to each language in addition to having heterogenous linguistic, cultural, and cognitive experiences (Koch et al., 2025). This differential exposure affects their performance on language and literacy assessments, which can make it challenging to determine their true linguistic abilities without considering both their languages (Gray et al., 2018; Ortiz & Cehelyk, 2023). Furthermore, different instructional programs can also affect their language and literacy performance and development (Bialystok, 2018). Francis et al. (2019) reported substantial variability in student performance in English and Spanish oral language and reading for students in dual language compared to English-only instruction. The authors concluded that language of instruction was a “non-ignorable factor” in evaluation of performance and classification of risk.

Substantial heterogeneity among bilingual students also impacts the choice of assessment; factors such as the language, normative sample, cultural appropriateness, reliability, and validity of the assessments are crucial indicators to consider to minimize bias and prevent potential harm from discriminatory testing practices (Brookhart & McMillan, 2020; Ortiz & Cehelyk, 2023). However, the challenge remains significant. For example, assessments often are not available in the student’s home language. Even when they are, they might still be normed primarily on monolingual speakers in other countries. Additionally, most assessments of English abilities have been developed and normed primarily for English monolingual speakers. Thus, there is a need to identify early screening measures that will accurately classify risk for reading difficulties in multilingual settings.

Unfortunately, there is currently a paucity of research on assessments that can accurately classify bilingual children at risk for reading difficulties. One study (Rhinehart & Gotlieb, 2023) of 54 first- grade students (41% English learners; some of whom were enrolled in dual language instructional programs) indicated that a dyslexia screener (Early Bird) appeared to perform similarly for English learners and non-English learners. However, despite reporting non-significant differences in the rate of students identified as at risk for dyslexia across groups, this study did not specifically evaluate whether the screening tool had adequate sensitivity or specificity for classifying bilingual students as at risk. Another study (Landry et al., 2022) reported that end of kindergarten scores derived from an early reading screener (Acadience Reading) were similarly predictive of long-term reading outcomes for both native English-speaking and English learner students (English learners were enrolled in dual language instructional programs). However, end of kindergarten scores did not meet thresholds for acceptable sensitivity and specificity for predicting future risk for reading difficulty (e.g., for English learners well below benchmark at end of kindergarten: sensitivity = 0.59 and specificity = 0.90). Thus, additional research is needed to identify early assessments of literacy-related skills that are strong predictors of future risk for reading difficulty among bilingual students in different instructional contexts.

## Early Screening in a Bilingual Context: Decisions

### Defining Reading Difficulty

When developing, implementing, and evaluating screening solutions to detect early reading difficulties, it is crucial to select the appropriate reading outcome and relevant predictors. Additionally, in a bilingual population, the language of assessment and the methodology for integrating scores across different languages require careful consideration. Reading performance can be measured in a variety of ways (e.g., oral reading fluency, nonword reading, reading comprehension), and the outcome selection can influence the classification and interpretation of results. In this study, we employ the Simple View of Reading framework, which defines ‘reading ability’ as a combination of decoding and linguistic comprehension skills (Hoover & Gough, 1990); therefore, our reading outcome assesses both decoding and comprehension skills. For bilingual children, the language of assessment also introduces different possibilities for determining the outcome: based on a composite score of English and Spanish performance, performance in the “best” language, or performance in the language of instruction. Underlying reading difficulties that are not due to lack of instructional opportunities should affect performance in both languages spoken by a child. Therefore, we chose to define reading difficulty based on low scores in children’s “best language.” For example, a child who had a standardized reading score two standard deviations below the mean in English might typically be considered “at risk” for a reading difficulty, but if this same child also had a standardized Spanish reading score within the typical range for their age (e.g., within one standard deviation of the normative mean), we would not consider this child to be “at risk.” In contrast, a child with scores more than one standard deviation below the mean in *both* Spanish and English might be considered “at risk” in this “best language” approach. This approach avoids issues of low scores in one specific language due to lack of exposure or low proficiency (potentially related to language of instruction). It also avoids problems that may emerge from computing an average or composite score across languages, for which unbalanced proficiency resulting in an extreme low score in one language could result in identification of a reading difficulty when a child has typical reading skills in their other language.

### Identifying Relevant Screening Targets

In addition to defining what constitutes an underlying reading difficulty, it is equally important to identify relevant early (i.e., preschool and kindergarten) predictors of reading performance in bilingual children. Studies with monolingual English students have found that skills like phonological awareness, letter identification, rapid automatized naming, and sentence repetition are relevant predictors of later reading performance (Catts et al., 2015; Silverman et al., 2021). Similarly, studies with bilingual students have found that some of these predictors are consistent across linguistic backgrounds. For instance, ‘language-general’ skills such as phonological awareness may transfer across languages, thus facilitating effective reading development in various linguistic contexts (Khalaf et al., 2019). A recent study by Giguere et al. (2024) found that Spanish phonological awareness and concepts about print predict English decoding skills. However, other skills, like vocabulary, might be more language-specific and tend to predict outcomes more strongly within a particular language, but not across languages (Melby-Lervåg & Lervåg, 2011). In fact, some research suggests that cross-linguistic transfer is in general weaker for oral language skills compared to phonological skills (Davison et al., 2011; Kremin et al., 2019; Lindsey et al., 2003; Manis et al., 2004; Melby‐Lervåg, & Lervåg, 2011). Despite this, oral language tasks—which are often omitted from early literacy screening batteries for monolingual students (e.g., January & Klingbeil, 2020)—could be especially valuable for bilingual kindergarteners who may not yet have significant exposure to English literacy instruction or sufficient English proficiency to demonstrate responsiveness to instruction. For these students, assessing their oral language in both L1 and L2 could offer valuable information when predicting future reading performance.

In Spanish-English bilinguals, early literacy-related skills, including phonological awareness, rapid automatized naming, and letter identification have been found to predict later reading abilities both within and across languages (Baker et al., 2022; Durgunoğlu et al., 1993; Lindsey et al., 2003; Kremin et al., 2019). Early oral language abilities have also been found to be important to reading outcomes: both semantic (e.g., vocabulary, knowledge of relations between words), and morphosyntactic/discourse skills (e.g., sentence repetition, oral comprehension, oral narrative measures) predict reading outcomes within and across languages (Bedore et al., 2023; Davison et al., 2011; Miller et al., 2006; Hammer et al., 2007; Rinaldi & Páez, 2008). In summary, both early literacy and oral language skills in each of the languages a child knows are important for reading development in bilingual children. However, few prior studies have examined the utility of screening measures other than English-only measures for predicting risk of reading difficulties in Spanish-English bilingual children, and of these none have explored whether considering scores across both Spanish and English oral language and early literacy screening measures (e.g., best language scores for a given construct, such as vocabulary knowledge) improves accuracy.

Petersen and Gillam (2013) examined the predictive accuracy of kindergarten language and early literacy measures in English and Spanish for identifying risk of reading difficulty in English at the end of first grade. Using a combination of best language scores of the kindergarten measures in a discriminant function analysis, they reported high specificity but generally inadequate sensitivity. However, this study included a relatively small sample of children who were at risk of language difficulty and focused solely on English reading as the outcome of interest. Therefore, it is unclear whether these results would generalize to a broader sample of children in typical universal screening settings. In a larger sample, Lindsey and colleagues (2003) found that kindergarten language and literacy measures administered in Spanish were significant predictors of language and reading outcomes in both English and Spanish. However, both sensitivity and specificity were inadequate (i.e., less than 0.80). Neither of these studies accounted for *both* English and Spanish measures as predictors and outcomes.

Two recent studies have investigated both Spanish and English measures as indicators of reading risk. Baker et al. (2022) examined the classification accuracy of Spanish and English literacy measures administered at three time points in Grades 1 and 2 for predicting separate Spanish and English reading comprehension outcomes at the end of the same school year. Participants included Spanish-English bilingual children in bilingual educational contexts. For each screener, six ROC analyses were performed. For both Spanish and English reading comprehension, three levels of risk were evaluated: at risk (20th percentile), some risk (40th percentile) and at target (60th percentile). Results revealed that the literacy measures (including letter naming, phonemic awareness, decoding, and reading fluency) largely met minimum acceptable accuracy (i.e., AUC greater than 0.75) when used as isolated predictors of reading risk in the same language, with some exceptions (e.g., phonemic awareness did not predict reading comprehension outcomes). Moreover, Spanish screening measures predicted English reading outcomes, but English screening measures did not predict Spanish reading outcomes. This study did not include oral language measures as screening targets and evaluated reading outcomes separately across languages, rather than creating a single indicator of reading achievement. Finally, Bedore et al. (2023) used oral language (semantics and morphosyntax) measures as screeners for reading risk in the fall of Grade 1. Students were Spanish-English bilingual children in dual language transition-to-English programs, with proportion of English and Spanish instruction varying by teacher. Using logistic regression, they reported that language risk (i.e., at or below the 25th percentile based on students’ best performance across languages) in semantics, but not morphosyntax, was associated with increased reading risk. However, the rate of reading risk identified in this sample (about 7%) was much lower than what would be expected based on the 25th percentile cutoff, suggesting this sample of children had higher overall reading scores than the normative sample and results may therefore not generalize to other samples. Additionally, this study did not include other potential screeners of risk for reading difficulty, such as measures of code-based early literacy skills (e.g., phonemic awareness).

## Current Study

Relevant prior research suggests that, independently, early literacy measures (e.g., letter naming, phonological awareness) and oral language assessments (e.g., sentence repetition, vocabulary) may effectively predict whether Spanish-English bilingual students are at risk of reading difficulties. However, two primary gaps in the literature remain. We attempted to address these gaps by first exploring the utility of *both* language and early literacy measures in English *and* Spanish for predicting future reading risk in a student’s strongest language of performance. Then, we evaluated whether a combination of measures (i.e., multivariate screening) increases classification accuracy. While several studies with monolingual students have explored this question and reported mixed results (e.g., Johnson et al., 2009; White & Schatschneider, 2023), there has been less research examining whether using multiple screening measures improves classification accuracy for bilingual students.

Because opportunities to respond to instruction are critical to identifying whether children are at risk for or have an underlying reading difficulty (Miciak & Fletcher, 2020) and language of instruction is a non-ignorable factor for assessing bilingual children’s academic skill development (Francis et al., 2019; Wackerle-Hollman et al., 2022), we analyzed data separately for students enrolled in dual-language instructional programs and English-only instructional programs. Different criteria for establishing risk for reading difficulty are likely needed across various instructional models. Dual-language models provide opportunities to respond to literacy instruction in whichever language children are most proficient, whereas many students may not respond to instruction in an English-only program due to relatively low English proficiency and thus limited access to reading instruction. We evaluated the following research questions.

### RQ1

*What is the classification accuracy of individual kindergarten oral language and early literacy measures for predicting risk for reading difficulty at the end of first grade?* We hypothesized that measures that tap transferable language skills (e.g., phonological awareness) would provide greater classification accuracy than measures that tap relatively language-specific skills (e.g., vocabulary knowledge). However, we expected that no single kindergarten measure would provide adequate classification accuracy (i.e., sensitivity of at least 0.90 and specificity of at least 0.80). We expected that considering children’s best score regardless of language for each construct (e.g., phonological awareness) would result in greater classification accuracy than would be obtained using only English or only Spanish scores.

### RQ2

*Does a multivariate approach to screening improve classification accuracy over individual measures?* To evaluate this research question, we used a multiple logistic regression approach with all measured constructs included in the model. Because this approach maximizes the information available in the classification (at the cost of being less accessible to practitioners), we expected that it would provide the greatest classification accuracy.

## Method

### Transparency and Openness

This study’s design and analysis plan was preregistered prospectively, before data were collected (https://osf.io/ufzkv/?view_only=c627688f2e1846fe807761f07e02b439). Because this study was not designed with questions regarding language of instruction in mind, we deviated from the design and analysis plan when we decided to analyze the data separately based on language of instruction. Moreover, we deviated from our original plan for models that included multiple screening measures, to shift towards a logistic regression approach. Data used in this analysis are available upon request.

### Participants

This study was reviewed and approved by the University of South Carolina Institutional Review Board. Schools were recruited for this project using a convenience sampling approach. Project recruitment took place during Fall 2021, when many schools were still not allowing external personnel into facilities due to concerns regarding the ongoing COVID-19 pandemic. Consequently, the principal investigators at the Texas and South Carolina sites approached local school districts who were proximal to the research teams regarding interest in participating in the study. Once district approval was obtained, principals of individual schools that enrolled relatively large percentages of Spanish-English bilingual children (relative to other schools in the districts) were approached regarding interest in participation. Upon agreement from principals, opt-out consent forms approved by the IRB were sent home to families of all Spanish-English bilingual kindergarten children enrolled in the schools. These forms notified caregivers of the nature of the research and provided an opportunity to either return the forms indicating they did not want their child to participate, or to contact the research team to ask questions and indicate they did not want their child to participate. Because this study was approved to use a passive consent process, all Spanish-English bilingual kindergarteners at participating schools were included in the sample, unless their caregivers had indicated they did not wish for the child to participate. Therefore, every eligible bilingual child at participating schools had an equal likelihood of participation.

Two-hundred twenty Spanish-English bilingual children across Texas and South Carolina participated in this project. All children were in kindergarten at the time of enrollment and were followed longitudinally through first grade. Children were identified as Spanish-English bilingual first through school report of language use in the home, which was subsequently confirmed by parent report and/or performance on kindergarten oral language assessments. We used an inclusive definition of bilingualism in which any child who was reported by the school and/or parents as being exposed to Spanish at home and who correctly answered at least one question on both Spanish and English expressive language assessments in kindergarten was eligible. This definition was used to ensure that we captured the full range of bilingualism and relative levels of proficiency in English and Spanish.

Approximately 56% percent of children were enrolled in dual-language instructional programs, all of which used a 50/50 Spanish-English instructional model (*n* = 123; all children in dual language instruction were enrolled at the Texas site). Three out of four schools used a two-way dual language model (i.e., approximately 50% of students with Spanish as the primary language and 50% of students with English as the primary language enrolled in the same classroom), and the fourth school used a one-way dual language model (all students in the classroom came from homes in which Spanish was the primary language). The remainder of the participants were enrolled in English-only instruction (*n* = 97; most children in English-only instruction were enrolled at the South Carolina site, with only one Texas school using English-only instruction) with additional supports for students identified as having limited English proficiency. Across instructional programs, child age (dual-language instruction *M* = 71.43 months, *SD* = 3.96 months; English-only instruction *M* = 73.02 months, *SD* = 3.91 months) and sex (dual-language instruction = 52.8% female; English-only instruction = 49.5% female) were similar.

### Measures

#### Kindergarten Oral Language

Children completed measures of English and Spanish oral language skills in kindergarten. To measure vocabulary knowledge, children completed the Expressive One-Word Picture Vocabulary Test—Spanish Bilingual Edition (EOWPVT-SBE; Martin, 2013). This measure is designed to assess bilingual children’s conceptual vocabulary knowledge (i.e., the number of concepts a child knows a word for, regardless of whether the word is known in English, Spanish, or both languages). However, given evidence that conceptual vocabulary scores are biased against bilingual children with relatively balanced proficiency across languages (Fitton et al., 2023), we chose to administer the assessment separately in English and Spanish, which allowed us to compute a conceptual vocabulary score as well as obtain specific English and Spanish vocabulary scores. Internal consistency reliability for this task is high for kindergarten-age children (α = 0.96). This assessment was normed on a sample of Spanish-English bilinguals. Children also completed the English and Spanish Sentence Repetition subtests of the Bilingual English-Spanish Assessment (BESA; Peña et al., 2014). This task is designed to measure children’s morphosyntactic (i.e., grammatical) knowledge. For this task, children must repeat sentences verbatim, and each item is scored for specific grammatical features. The Spanish version of the assessment contains 10 sentences to be repeated, and the English version of the assessment contains 9 sentences to be repeated. Internal consistency reliability for this subtest is high in both English and Spanish (α ranges from 0.95 to 0.96). This assessment was normed on a sample of Spanish-English bilinguals.

#### Kindergarten Early Literacy

In addition to measures of oral language proficiency, children completed measures of kindergarten early literacy skills. To measure instant letter recognition, children completed the Rapid Letter Naming subtest of the Comprehensive Test of Phonological Processing (CTOPP-2; Wagner et al., 2013) and its Spanish equivalent, the Test of Phonological Processing in Spanish (TOPPS; Francis et al., 2001). For each of these tests, children viewed an array of 36 letters and were asked to name the letters as quickly and accurately as possible. Prior to completing the task, children were asked to name the six letters included on the task as a practice task to ensure they knew the letters. If children were unable to name the letters, the examiner attempted to briefly teach the letter names. If children still could not name the letters, the test was discontinued. The total number of errors that children made and the number of seconds taken to complete the task were recorded. The time taken to complete the task was used to obtain scale scores, except for instances in which the child made more than 4 errors on the assessment. Test-retest reliability for the Rapid Letter Naming subtest of the CTOPP-2 is high (*r* =.85). The CTOPP-2 was normed on a sample of primarily monolingual English speakers. Reliability information for the TOPPS is not available, as this measure is not published or normed. Because administration procedures were identical across languages, scale scores (mean of 10 and standard deviation of 3) for the TOPPS Rapid Letter Naming subtest were derived from the CTOPP-2. Prior research indicates that the CTOPP-2 demonstrates sufficient evidence of measurement invariance across Spanish-English bilingual and monolingual English-speaking children (Shergill et al., 2023).

To measure phonological/phonemic awareness, children completed the Elision subtest of the CTOPP-2 and TOPPS. For this test, children were presented with a word orally and asked to remove part of the word to create a new word (e.g., *say “cat” without /k/*). The tests begin with items that require manipulation of large phonological units and progressively become more difficult until children are asked to manipulate individual phonemes. Internal consistency reliability for the Elision subtest of the CTOPP-2 is high for kindergarten-age students (α ranges from 0.90 to 0.92). Reliability information for the TOPPS Elision subtest is not available. Scale scores could not be derived for TOPPS Elision because of differences in number of items and administration procedures across the TOPPS and CTOPP-2.

#### First Grade Reading

To measure reading skills in first grade, children completed three subtests of the Woodcock-Johnson IV Tests of Achievement (WJ-IV; Schrank et al., 2014) and the parallel Batería IV Woodcock-Muñoz Pruebas de Aprovechamiento (Batería IV; Woodcock et al., 2019). Children completed the Letter-Word Identification (Identificación de Letras y Palabras), Word Attack (Análisis de Palabras), and Passage Comprehension (Comprensión de Textos) subtests. The Letter-Word Identification subtest begins with simple items that assess knowledge of letter names and sounds and progresses in difficulty to requiring children to read increasingly difficult lists of real words. The Word Attack subtest is similar in structure to Letter-Word Identification but asks children to read increasingly difficult lists of nonsense words. The Passage Comprehension subtest begins with items that ask children to read individual words and point to a picture corresponding to the words and progresses to more difficult items that require children to read sentences or short paragraphs and provide a missing word. Internal consistency reliability of these subtests is high for first grade children (α ranges from 0.93 to 0.98). Standard scores for the WJ-IV and Batería IV are derived from norming samples of primarily monolingual individuals. Specific data for reliability and validity for bilingual students are not available (Braden, 2024).

### Procedure

This study was approved by the Institutional Review Board of Texas A&M University and the University of South Carolina. Informed consent was provided for all children prior to enrollment in the study. Children completed all assessments in a quiet area of their school with trained bilingual research assistants. The kindergarten assessment battery was completed during the second half of the kindergarten school year and first grade assessments were completed approximately one year later, during the second half of the participants’ first grade school year. Assessments in Spanish and English were conducted on separate days, and the order of administration varied across children. Assessment sessions lasted approximately 30 min and were discontinued if children began to show signs of fatigue. Children received a small prize (e.g., sticker) for their participation in each assessment session, regardless of whether they finished all assessments planned for that session. During the data collection process, there were several instances in which children were unable to complete the Rapid Letter Naming subtest in one or both languages, especially for the Spanish version of the test when children were enrolled in English-only instructional programs. To minimize missing data, children who could not complete the Rapid Letter Naming subtest were assigned a scale score of 0, and children who passed the practice items but made more than 4 errors received a scale score of 1.

### Operationalizing Risk for Reading Difficulty

Prior to conducting individual ROC curve analyses, we needed to define risk for reading difficulty for students in English-only and dual-language instructional programs. Factor analyses of the word reading measures indicated that Spanish and English reading subtests are best represented as separate but related factors, as opposed to a unidimensional construct: c^2^(8) = 43.85, *p* <.001; CFI = 0.976; TLI = 0.955; RMSEA = 0.144; 0.039 . Each of the three first grade reading subtests (Letter-Word Identification, Word Attack, Passage Comprehension) yielded comparable loadings onto their respective language factor. Accordingly, to operationalize risk for reading difficulty in a way that is replicable in school settings, we first computed a composite (average) score from the standardized scores obtained from each of the three subtests within each language. Then, to avoid overidentification due to lack of exposure to reading instruction or low proficiency in one language, we chose the child’s best composite reading score, regardless of which language it was obtained in. Thus, if a child has strong reading skills in *either* language, they would not be categorized as at risk for reading difficulty.

Based on this “best language” reading variable, we dichotomized the data at three separate cut points that are frequently used to represent risk for various cognitive difficulties: one standard deviation below the mean, 1.5 standard deviations below the mean, and two standard deviations below the mean. Because the reading assessments were normed on monolingual individuals and instructional opportunities were not always provided in the language children were most proficient in, we expected that a larger percentage of our sample would fall below these cut scores than would be seen in the general population (e.g., 16% of individuals scoring more than one standard deviation below the mean in a normal distribution). Therefore, we examined the base rates of risk for reading difficulty by instructional model at each cutoff. Our goal was to use a cutoff of risk for reading difficulty that approximated school-based response-to-intervention models, in which it is common for 20% of students to be identified as needing more intensive supports than what is provided by general classroom instruction (Fletcher & Vaughn, 2009; Vaughn et al., 2006). In addition, exploring different cut points helped us better understand the risk classifications for reading difficulty in this bilingual sample compared to the monolingual norms, beyond relying solely on traditional sample-based cut scores.

### Data Analytic Plan

To address the primary research questions of the project, we used ROC curve analysis. This analysis is used to determine the classification function of a screening measure (in this study, kindergarten oral language or early literacy measures) for predicting a binary outcome (in this study, risk for reading difficulty defined based on best-language performance on first grade reading measures). This analysis provides indices of overall classification accuracy (e.g., area under the curve [AUC]), as well as more specific indicators of how accurately the screening measure classified different students. For this study, we focus primarily on sensitivity and specificity as described in the literature review. Although some research suggests that sensitivity and specificity of 0.80 represent acceptable values, we chose to set a minimum threshold of acceptability for sensitivity at 0.90 to minimize the number of students who were at risk for reading difficulties in first grade but were not identified by the screener (i.e., false negatives). We then evaluated the performance, in terms of specificity, of each individual kindergarten measure (as well as the “best language” scores for each measure; RQ1) and the performance of combinations of measures (RQ2). All analyses were conducted separately by language of instruction.

To address RQ1, we first conducted a ROC curve analysis for each measure of kindergarten oral language and early literacy skills (e.g., Spanish vocabulary, English vocabulary, Spanish phonological awareness, English phonological awareness). Then, we computed a variable representing the best score for each construct^1^ (e.g., the child’s best vocabulary score, regardless of the language it was obtained in). We conducted ROC curve analyses for each “best” language construct. For RQ2, we conducted three separate logistic regression analyses predicting risk of reading difficulty in first grade using: (1) Spanish kindergarten predictors, (2) English kindergarten predictors, (3) “best language” kindergarten predictors. Statistical significance of individual predictors is reported alongside effect size metrics (odds ratios [OR]). Predicted probabilities from each of these models were generated and used in a subsequent ROC curve analysis to determine whether any of the three multivariate approaches improved classification accuracy for predicting risk for reading difficulty in first grade.

## Results

### Descriptive Statistics and Preliminary Analysis

Average scores of kindergarten and first grade measures are reported separately for children in English-only and dual language instruction in Table 1 (kindergarten oral language and early literacy variables are reported in the upper panel, first grade reading outcomes are reported in the lower panel). For children enrolled in English-only instruction, Spanish kindergarten oral language and early literacy skills were consistently more than a full standard deviation below the mean (with floor effects present in the data for Spanish early literacy skills). Similarly, Spanish first grade reading outcomes were approximately two standard deviations below the normative mean. In contrast, English vocabulary knowledge and rapid letter naming scores were in the average range, with English sentence repetition and phonological awareness below average. First grade English reading outcomes were within the average range. For children enrolled in dual-language instructional programs, mean kindergarten oral language and early literacy skills were in the average range for all skills (though early literacy skills were consistently at the low end of the average range). First grade reading outcomes were all in the average range. Scores were relatively similar across languages, with somewhat greater word reading skills in Spanish than in English.

**Table 1:** Descriptive statistics for students in English-only and dual-language instructional programs

	English-Only Instruction		Dual-Language Instruction	
	Mean	SD	Mean	SD
Spn Vocab^a^	74.03	19.23	95.03	16.81
Eng Vocab^a^	96.89	18.66	92.42	23.50
Con Vocab^a^	104.25	12.43	108.91	15.10
Spn Sent Rep^a^	75.16	16.61	91.89	15.95
Eng Sent Rep^a^	79.86	18.01	86.30	20.24
Spn PA^b^	1.58	2.92	3.45	4.55
Eng PA^c^	6.62	3.22	7.15	3.60
Spn RLN^c^	0.42	1.81	7.28	4.65
Eng RLN^c^	8.09	4.32	7.13	4.80
Spn LWID^a^	73.98	21.69	111.07	25.27
Eng LWID^a^	88.26	19.83	95.86	22.76
Spn Word Att^a^	69.00	21.34	103.68	28.02
Eng Word Att^a^	93.11	18.78	103.39	20.29
Spn Pass Comp^a^	68.33	12.61	90.64	14.98
Eng Pass Comp^a^	86.90	14.34	90.39	15.62

Note. English-only *n* = 97. Dual-language (Spanish and English instruction) *n* = 123

*Spn* Spanish, *Eng* English, *Con* Conceptual, *Vocab* Vocabulary, *Sent Rep* Sentence Repetition, *PA* Phonological awareness, *RLN* Rapid letter naming, Letter-Word Identification *WordAtt* Word Attack, *Pass Comp* Passage Comprehension

^a^Means provided represent standard scores (mean for each age in norming sample = 100, standard deviation = 15)

^b^Means provided represent raw scores

^c^Means provided represent scale scores (mean for each age in norming sample = 10, standard deviation = 3)

The percentage of children receiving their highest score in Spanish and English for each construct is reported in Table 2. This is particularly relevant for characterizing the reading outcome, because we used children’s “best language” score to categorize risk for reading difficulty. As can be seen in Table 2, there were major differences in the language in which children received their best language reading score across instructional contexts. Nearly all (92.8%) children in English-only instruction had a higher standardized reading composite score in English than in Spanish. In contrast, approximately two thirds of children in dual-language contexts had a higher standardized reading composite score in Spanish than in English.

**Table 2:** Percentages of “best language” scores by construct

	English-Only			Dual Language		
	Spanish	English	Equal^a^	Spanish	English	Equal^a^
Kindergarten						
Sent. Rep.	44.0	52.7	3.3	56.5	35.7	7.8
Vocabulary	27.5	72.5	0.0	56.0	42.3	1.7
Letter Naming	0.0	90.2	9.8	41.3	35.6	23.1
PA	31.2	35.5	33.3	33.7	44.2	22.1
First Grade						
Reading	7.2	92.8	0.0	65.9	34.1	0.0

Note. English-only *n* = 97. Dual-language (Spanish and English instruction) *n* = 123

*Sent Rep* Sentence Repetition, *PA* Phonological awareness

^a^Equal scores indicates the scores were exactly equivalent across languages (e.g., Sentence Repetition scale score of 10 in both languages). No significance testing was done in determination of best language scores

### ROC Curve Analysis

#### Cut Points and Base Rates for Reading Difficulty Risk

For children in English-only instruction, 42.3% of students were classified as at risk when using the least stringent criteria for risk (best language reading composite score more than one standard deviation below the mean). When using 1.5 standard deviations below the mean, the base rate of risk was reduced to 23.7%. When using two standard deviations below the mean, the base rate of risk was further reduced to 10.3%. Accordingly, we selected 1.5 standard deviations below the mean as the cut point for identifying students who were in English-only instruction as being at risk for reading difficulty. This cutoff provided a base rate most closely aligned with the percent of students in school settings who receive supplemental support for developing reading skills in a response-to-intervention framework (around 20%, Fletcher & Vaughn, 2009; Vaughn et al., 2006).

For children in dual language instruction, the one standard deviation below the mean cut point provided a base rate of risk of 17.9%. This was reduced to 13% when using 1.5 standard deviations below the mean and further reduced to 6.5% when using two standard deviations below the mean. Therefore, for dual-language instruction, we classified students as at risk for reading difficulty if their best language reading composite score was more than one standard deviation below the normative mean.

#### English-Only Instruction

Results for children in English-only instruction are reported in Table 3. To evaluate RQ1, all kindergarten oral language and early literacy variables were examined in ROC curve analyses individually. Spanish oral language skills provided poor classification accuracy, as use of both sentence repetition (AUC = 0.35, *p* <.05) and vocabulary (AUC = 0.32, *p* <.05) standard scores as screening measures resulted in classification accuracy that was below chance. Classification accuracy of Spanish early literacy measures was equivalent to chance (for phonological awareness AUC = 0.51, *p* =.84; for rapid letter naming AUC = 0.55, *p* =.48). In contrast, English oral language and early literacy skills all had classification accuracy significantly greater than chance (all *p*s < 0.001; for sentence repetition AUC = 0.76; for vocabulary AUC = 0.81; for phonological awareness AUC = 0.76; for rapid letter naming AUC = 0.76). Classification accuracy of conceptual vocabulary scores was also statistically better than chance but did not reach acceptable levels (AUC = 0.67, *p* <.05). Best language sentence repetition scores had classification accuracy equivalent to chance (AUC = 0.55, *p* =.46), whereas best language vocabulary had significantly better than chance (but below acceptable) classification (AUC = 0.67, *p* <.05). Best language rapid letter naming (AUC = 0.76, *p* <.001) and phonological awareness (AUC = 0.66, *p* <.05) demonstrated significantly better than chance classification.

**Table 3:** Results of ROC curve analysis for children in English-only instructional programs.

Model	AUC	Sensitivity (when set to 0.90 or higher)	Specificity
Individual Screeners			
Spn Sentence Repetition	0.35^*^	1.00	0.00
Spn Vocabulary	0.32^*^	0.96	0.03
Spn Letter Naming	.55^ns^	0.96	0.14
Spn Phonological Awareness	.51^ns^	0.96	0.07
Eng Sentence Repetition	0.76^***^	0.91	0.49
Eng Vocabulary	0.81^***^	0.91	0.55
Eng Letter Naming	0.76^***^	1.00	0.17
Eng Phonological Awareness	0.76^***^	0.91	0.39
Conceptual Vocabulary	0.67^*^	0.91	0.46
Best Sentence Repetition	.55^ns^	1.00	0.01
Best Vocabulary	0.67^*^	0.91	0.51
Best Letter Naming	0.76^***^	1.00	0.16
Best Phonological Awareness	0.66^*^	0.96	0.16
Logistic Regression			
Spanish Predictors	0.73^**^	0.96	0.25
English Predictors	0.83^***^	0.91	0.54
Best Language Predictors	0.79^***^	0.96	0.46

Note. *N* = 97. *Spn* Spanish, *Eng* English, *AUC* Area under the curve

^***^ *p* <.001. ^*^ *p* <.05. ^ns^ = non-significant

Based on our a priori criterion for classification accuracy thresholds, the best kindergarten predictor of risk for reading difficulty in first grade among children in English-only instruction was English vocabulary knowledge. The optimal cut score had sensitivity of 0.91 and specificity of 0.55, indicating that restricting false negatives to 9% resulted in 45% of positive screening results being false positives (children not at risk for reading difficulties inaccurately flagged as being at risk). Overall classification accuracy was 63.5%.

To address RQ2, we conducted three logistic regression models. The first model included all kindergarten Spanish measures as predictors of risk for reading difficulty in first grade. No measures were significant, unique predictors of risk for reading difficulty. When predicted probabilities from this model were used as the primary screener in the ROC analysis, overall classification accuracy was significantly greater than chance (AUC = 0.73, *p* <.01). The second model included all kindergarten English measures as predictors of risk for reading difficulty in first grade. Only English rapid letter naming was a significant predictor, with higher English rapid letter naming scores associated with decreased risk for reading difficulty (OR = 0.86, *p* <.05). When predicted probabilities from this model were used as the primary screener in the ROC analysis, overall classification accuracy was significantly greater than chance (AUC = 0.83, *p* <.001). The third model included kindergarten “best language” measures as indicators of risk for reading difficulty in first grade. Results of this model indicated that best language rapid letter naming (OR = 0.83, *p* <.01) was the only significant, unique predictor of risk for reading difficulty in first grade. When predicted probabilities from this model were used as the primary screener in the ROC analysis, overall classification accuracy was significantly greater than chance (AUC = 0.79, *p* <.001). However, no multivariate model exceeded the balance of sensitivity and specificity achieved when using English vocabulary scores alone (see Table 3).

#### Dual-Language Instruction

Results of analyses for children in dual language instruction are reported in Table 4. When evaluating RQ1, Spanish oral language skills (sentence repetition AUC = 0.63; vocabulary AUC = 0.50) did not provide better than chance classification. All other oral language and early literacy indicators in Spanish and English provided classification that was significantly better than chance (AUCs range from 0.70 to 0.89). However, only English early literacy skills approached acceptable specificity levels when sensitivity was restricted to 0.90 or higher, with English rapid letter naming scores providing the strongest classification accuracy (AUC = 0.89, sensitivity = 0.96, specificity = 0.76). When examining best language scores, all measures provided significantly better than chance classification (AUCs range from 0.66 to 0.85); however, no best language models had better sensitivity and specificity than the model with English rapid letter naming as the screener. In that model, restricting false negatives to 4% resulted in 24% of students who were not at risk of reading difficulty in first grade being incorrectly flagged as at risk in kindergarten (false positives). This yielded an overall classification accuracy of 79.6%.

**Table 4:** Results of ROC curve analysis for children in dual-language instructional programs.

Model	AUC	Sensitivity (when set to 0.90 or higher)	Specificity
Individual Screeners			
Spn Sentence Repetition	.63^ns^	0.96	0.06
Spn Vocabulary	.50^ns^	0.96	0.08
Spn Letter Naming	0.80^***^	0.96	0.42
Spn Phonological Awareness	0.76^***^	0.96	0.38
Eng Sentence Repetition	0.74^***^	0.96	0.25
Eng Vocabulary	0.79^***^	0.95	0.38
Eng Letter Naming	0.89^***^	0.96	0.76
Eng Phonological Awareness	0.79^***^	0.91	0.68
Conceptual Vocabulary	0.70^**^	0.95	0.26
Best Sentence Repetition	0.71^**^	0.95	0.16
Best Vocabulary	0.66^*^	0.90	0.27
Best Letter Naming	0.85^***^	0.91	0.63
Best Phonological Awareness	0.80^***^	0.91	0.47
Logistic Regression			
Spanish Predictors	0.88^***^	0.91	0.82
English Predictors	0.94^***^	0.95	0.80
Best Predictors	0.92^***^	0.94	0.77

Note. *N* = 123. *Spn* Spanish, *Eng* English, *AUC* Area under the curve

^***^ *p* <.001. ^**^ *p* <.01. ^ns^ = non-significant

To address RQ2, we conducted three logistic regression models (see lower panel of Table 4). The first model included all kindergarten Spanish measures as predictors of risk for reading difficulty in first grade. All kindergarten Spanish measures were significant, unique predictors of risk for reading difficulty (Sentence Repetition OR = 0.94, *p* <.05; Vocabulary OR = 1.07, *p* <.05; Phonological Awareness OR = 0.71, *p* <.05; Rapid Letter Naming OR = 0.79, *p* <.01). For all measures except for Spanish vocabulary knowledge^2^, higher scores were associated with lower risk for reading difficulty in first grade. When predicted probabilities from this model were used as the primary screener in the ROC analysis, overall classification accuracy was significantly greater than chance (AUC = 0.88, *p* <.001). The second model included all kindergarten English measures as predictors of risk for reading difficulty in first grade. English rapid letter naming (OR = 0.68, *p* <.001) and phonological awareness (OR = 0.62, *p* <.05) in kindergarten were significant predictors, with higher scores associated with decreased risk for reading difficulty. When predicted probabilities from this model were used as the primary screener in the ROC analysis, overall classification accuracy was significantly greater than chance (AUC = 0.94, *p* <.001), and this model yielded the optimal balance of sensitivity (0.95) and specificity (0.80)^3^. The third model included kindergarten “best language” measures (and English and Spanish phonological awareness, separately) as indicators of risk for reading difficulty in first grade. Results of this model indicated that best language rapid letter naming scores (OR = 0.72, *p* <.001) and phonological awareness scores (OR = 0.97, *p* <.05) were significant, unique predictors of risk for reading difficulty in first grade. When predicted probabilities from this model were used as the primary screener in the ROC analysis, overall classification accuracy was significantly greater than chance (AUC = 0.92, *p* <.001). However, this model did not exceed the balance of sensitivity and specificity achieved either when using English rapid letter naming alone or when using all English scores in a multivariate model.

## Discussion

Overall, findings revealed substantial differences in classification accuracies obtained for Spanish-English speaking children enrolled in classrooms that used English-only instruction compared to those enrolled in Spanish-English dual language classrooms. All approaches for predicting risk for reading difficulty in first grade were more accurate for children in dual language compared to English-only instruction. For children receiving dual language instruction, kindergarten English rapid letter naming yielded relatively strong prediction of first grade reading risk as an individual measure, with an overall classification accuracy of 79.6% (with false negatives minimized to 4% and false positives at 24%). Conversely, for children receiving English-only instruction, the individual measure that yielded the highest classification accuracy was English vocabulary at 63.5% (with false negatives minimized to 9% and false positives at 45%). Considering children’s “best” score for each construct (i.e. sentence repetition, vocabulary, rapid letter naming), regardless of the language it was obtained in, resulted in reduced classification accuracy in most cases, with no instances of improved classification accuracy.

Contrary to our hypotheses, the multivariate approaches did not provide substantial improvement in classification accuracy for students in either instructional setting. Among children in English-only instruction, using either all measured English skills or all “best” scores for each construct, regardless of language, resulted in highly similar classification accuracy to what was obtained by measuring English vocabulary alone (e.g., false positive rate near 45% when false negative rate was 9%). These values were well below typical standards needed for effective screening practice^4^. For children enrolled in dual language instruction, utilizing information from all constructs measured in English did result in a small improvement in specificity over the model using only English rapid letter naming. However, given the increased time and cost of assessing every student on four skills, alongside the need to employ a technically intensive logistic regression technique to generate scores that can be used to determine which students are at risk for future reading difficulty, we do not believe that this improvement is substantial beyond what is obtained from a quick (less than 5 min) assessment of rapid letter naming.

### Identification of Reading Difficulty

A key factor in evaluating classification accuracy of screening practices is the quality of the criterion measure used to operationalize the outcome, in this case risk for reading difficulty. For the present study, we used three measures of reading skill, which were each administered in both Spanish and English to participating children during the second half of their first-grade school year. The selected measures have strong reported psychometric properties and are constructed similarly across Spanish and English (Schrank et al., 2014; Woodcock et al., 2019); however, as with most norm-referenced assessments, there is limited evidence informing their reliability or validity for measuring reading among bilingual individuals. This is a persistent challenge in bilingual language and reading research broadly, as there are few assessment tools designed to account for the complexities of bilingual oral language and/or reading development.

Cut points based on children’s best language reading score across Spanish and English were used to determine risk status, ensuring we did not categorize risk based solely on low performance in one language that may be due to factors other than an underlying reading disability (e.g., limited oral proficiency in that language). At times, use of the best language approach means that an individual screener will not be in the same language as some children’s best language reading outcome (e.g., for children whose best language reading score is in English, all Spanish screening measures will be in a different language). However, our aim was not to model crosslinguistic interactions or individual differences in reading skills as continuously distributed skills across languages. Rather, we focused on identifying measures that maximize the classification accuracy of overall risk for reading difficulty treated as a binary outcome. In a “best language” approach, students are considered at risk only if they demonstrate low reading performance in both languages, enabling us to define a reading risk outcome that is minimally influenced by other factors (e.g., alignment between child language proficiency and the language of instruction).

Future studies should explore other approaches to screening bilingual children for reading risk in English-only instructional contexts. For example, one recent study suggests that evaluating children’s performance on a screening assessment stratified by English proficiency levels may provide a more appropriate reference group (Marrs et al., 2022). Comparing bilingual children’s achievement to the achievement of other bilingual children represents an improvement over prior comparisons to the monolingual normative samples for which most standardized reading assessments are constructed. However, it may be important to narrow the reference group to which bilingual children are compared even further. For example, comparing reading achievement of bilingual children with Level 1 English proficiency to the achievement of other same-age bilingual children with Level 1 English proficiency in similar instructional contexts may improve prediction of reading risk for children in English-only instructional contexts. Other approaches to assessment should also be explored, such as dynamic assessment, which may provide a more accurate evaluation of a child’s learning potential and improve classification accuracy for children in English-only instruction (Petersen & Gillam, 2015; Vellutino et al., 1998).

### Differences by Language of Instruction

Prior research indicates that language of instruction is a non-ignorable factor when considering the measurement and interpretation of Spanish-English bilingual children’s reading development (Francis et al., 2019; Wackerle-Hollman et al., 2022). Our findings support this conclusion. We observed differences not only in participants’ language and reading performance by language of instruction, but also in the degree to which kindergarten screening measures predicted risk for reading difficulty in first grade. Classification accuracy was higher for children enrolled in dual language compared to English-only instruction.

As previously noted, there are multiple plausible explanations for the observed differences between groups. One is that the classroom language of instruction differentially impacts Spanish-English speaking children’s reading development. Specifically, the higher rate of students identified as at-risk in English-only classrooms may indicate that Tier 1 instruction is not optimally meeting these students’ academic needs. This is consistent with research showing that emergent bilinguals often benefit from literacy development in their first language (Collier & Thomas, 2017) and “students instructed in their native language as well as in English perform better, on average, on measures of English reading proficiency than language minority students instructed only in English” (August et al., 2000, p. 437). Dual language instruction may support bilingual children’s academic development more holistically than English-only instruction (e.g., Collier & Thomas, 2017; Farver et al., 2009). As of 2022, approximately 40% of Hispanic children between the ages of 3 to 4 years old attended public or private preschool prior to kindergarten enrollment (NCES, 2024). Although this number does not give an exact estimate of the number of Spanish-speaking children whose first exposure to English occurs in kindergarten, it does suggest that a substantial number of children from Spanish-speaking homes enter kindergarten with limited prior academic experience. Because the transition to kindergarten is widely recognized as a substantial step in children’s academic development, it can be both an impactful and stressful time for any child and their family (Mashburn et al., 2018; Purtell et al., 2020). When this transition co-occurs with the additional transition of being immersed in an unfamiliar or less-familiar language via English-only instruction, it may impact children’s capacity to learn. Even with evidence-based approaches in place to support bilingual students’ development of English proficiency, children may exhibit slower rates of growth in academic skills as they adjust to school and build capacity to learn in English (e.g., Fan, 2010).

Dual language instruction removes the need for an abrupt shift in language use that coincides with the transition into school (Fan, 2010; Slavin & Cheung, 2005). Further, it also has the potential to take advantage of any cross-linguistic transfer of literacy skills and concepts (e.g., Chung et al., 2019; Cummins, 1981). In the case of Spanish-English bilingualism, there is evidence that some literacy skills can be learned in one language and then transfer to the other, expediting overall learning across languages (e.g., Goodrich et al., 2013; Wawire & Kim, 2018). For example, many of the letter-sound correspondences that exist in Spanish are the same or similar in English. This foundational knowledge may support more rapid learning of the more complex English letter-sound correspondences.

One core limitation of the present work that precludes any strong conclusions about the differential impact of language of instruction is the near-perfect confound of language of instruction and site within the present study. All participants enrolled in dual language instruction were in Texas and most participants enrolled in English-only instruction were in South Carolina (though there was one Texas English-only school). Because this work was originally undertaken without an explicit plan to evaluate the impact of language of instruction, participant sampling was conducted based on the existing environments, rather than with an effort to obtain representation of both language models in each state. This means that any of the existing differences in the Texas and South Carolina environments cannot be disentangled from the impact of dual language versus English-only instructional approaches.

In the case of South Carolina, dual language instruction was rare in public schools during the years that this study was conducted. The South Carolina Spanish-speaking population is rapidly growing but remains a relatively small portion of the overall state population (8.4%; U.S. Census Bureau, 2024)). This is changing within individual schools and districts that have more concentrated numbers of bilingual students, with the result that more dual language programs have opened in recent years (Bilingualism Matters Center, 2024). However, there remains a lack of specialty training in multilingualism for educators, and there is a dearth of multilingual education professionals in the state (e.g., ASHA, 2021). In general, Spanish-speaking families can experience difficulty accessing information and navigating the public school system based on this broader environment, though there have been substantial advancements in the available supports for multilingual families in the state in recent years.

In contrast, the environment of Texas is different in that dual language instruction is far more common and support for Spanish-English bilingualism is more widespread. Many dual language instructional programs are in place to support Spanish-English speakers, and there is a closer match in the percentage of multilingual education professionals relative to the percentage of multilingual students in the state. For example, many pre-service teacher training programs in Texas have degree or certification options specifically focused on bilingual education, and a large portion of the Texas population speaks a language other than English at home (35.8%; U.S. Census Bureau, 2024). Consequently, many Texas colleges and universities serve a large number of Spanish-speaking students, resulting in a larger number of certified teachers who are proficient in both Spanish and English than in states with smaller Spanish-speaking populations, such as South Carolina.

Beyond the support for Spanish-English bilingualism by state, it is difficult to assess the potential impact of other differences between South Carolina and Texas. All children in the present sample were enrolled in Title I schools, suggesting relatively limited variation in socioeconomic status by site. Within Title I schools, there are likely children who come from homes with a range of different socioeconomic indicators, but overall Title I status indicates that the enrolled students are primarily from low-income homes. In considering the implementation of evidence-based practices to support literacy, we did not assess this directly within the participating schools but note that in general there was wide variation in the approaches to literacy instruction within the schools at the time of the study. In both South Carolina and Texas, all schools are required to use a curriculum that emphasizes phonics instruction, but the degree to which certain components of curricula are emphasized can vary across classrooms. Parent report of language use at home across sites indicated differences, with more Spanish used in the home for children at the Texas site; however, it is unclear whether the low percentage of Spanish use at home at the South Carolina site (~ 28% of input) accurately reflects children’s experiences or represents a certain amount of desirability bias in reporting (Cychosz et al., 2021). A more comprehensive evaluation of these environmental factors, and more strategic consideration of language of instruction, is needed to establish a clearer picture of how differences in state context and language of instruction impact bilingual students’ experiences and capacity to screen for risk for reading difficulty based on kindergarten language and reading performance.

### Additional Limitations

In addition to the confound between site and language of instruction, there are several additional limitations important to consider in relation to the findings. First, although the total sample size was reasonable for the primary study objectives, the subgroup analyses based on the language of instruction within the classroom were conducted based on substantially smaller samples. Fewer than 100 students in the study were enrolled in English-only instruction. This sample size limits our ability to detect small but potentially informative differences in classification function across the predictive models tested. Given the heterogeneity inherent to the bilingual population in the U.S., it is plausible to expect that if this study were completed with a larger sample of students, or with students in different regional contexts who speak different languages, the specific findings may change.

Another consideration is that the measures included in the present study are limited in the constructs that they represent. For example, our measure of kindergarten letter knowledge, which was the strongest indicator of risk for reading difficulty for children in dual language instruction, was a letter naming fluency task. Without a measure of total alphabet knowledge, it is difficult to disentangle whether it is variance due to automaticity of letter-name knowledge or variance due to cognitive processing speed that is driving accurate classification of reading risk. Similarly, we only have one measure of phonological awareness, an elision task, which was difficult for kindergarten children in this sample, especially in Spanish. Although we framed our research in the context of the simple view of reading and included a measure of reading comprehension, individual differences in scores on this task in first grade may primarily represent differences in children’s decoding skills. It is possible that different screeners would emerge as the best indicators of future risk if reading comprehension was measured more comprehensively and at later grades, after it becomes a more distinct construct that is independent of decoding.

Finally, it is important to consider the possibility that optimal prediction of risk for reading disability may not be feasible in kindergarten. Although increases in preschool enrollment have led to increases in reading readiness skills among some kindergarteners, many children still enter kindergarten with limited experience with reading. These differences in prior experience with literacy-related skills make it difficult to distinguish between children who are following typical patterns of literacy development and those who are experiencing difficulty acquiring literacy skills. For multilingual learners, these challenges are exacerbated when there are mismatches between the language(s) of classroom instruction and children’s oral language proficiency. It may be necessary for students to experience a longer period of consistent literacy instruction, and oral language development, before screening can be conducted with a reasonable level of accuracy, especially in relation to minimizing false positives. This does not mean that screening should not be conducted to start offering support to identified students, but rather that we may need to expect a higher rate of false positives when literacy screening is conducted in kindergarten compared to the later elementary grades.

## Conclusion

Overall, results from the present work suggest that, at least in the context of bilingual students in dual language classrooms, there is potential to screen effectively for later reading difficulty using brief measures administered during kindergarten. We found that English rapid letter naming yielded reasonable classification accuracy when minimizing false negatives was prioritized. This specific finding requires further exploration given the specific characteristics of the study sample, particularly given that all the dual language classrooms were located in Texas, and we have limited information about the core components of literacy instruction within those classrooms. It is likely that other measures, potentially in English and/or Spanish, may emerge as more effective screeners under different conditions. For children enrolled in English-only instruction from kindergarten, we did not find evidence that early screening for reading difficulty can be used to classify risk for reading difficulty one year later with optimal classification accuracy, sensitivity, and specificity. Even when multiple measures were included, classification accuracy did not approach acceptable levels for the English-only classrooms. The experience of Spanish-English bilingual learners within these contexts is complex and appears to impact performance strongly, at least during kindergarten. Further research is needed to better understand the complexities of these experiences and develop measurement techniques that more effectively distinguish children with underlying disabilities in reading early.

## Footnotes

1 Note, for phonological awareness, we computed sample-specific percentiles separately for children in English-only and dual-language instruction, as the scores across English and Spanish were not directly comparable (see Method section). These percentiles were used as children’s “best language” phonological awareness scores. For example, if a child was at the 50th percentile of the sample in English phonological awareness, but the 25th percentile for Spanish phonological awareness, the higher percentile (English) was used as their score.

2 The association between higher Spanish vocabulary and greater risk for reading difficulty likely represents a statistical suppression effect, as the zero-order point-biserial correlation between kindergarten Spanish vocabulary scores and first grade risk for reading difficulty is 0.00 and non-significant.

3 A slightly different cut score yielded sensitivity of 0.91 and specificity of 0.87, but we elected to use the cut score with the sensitivity that more closely matched that obtained using English rapid letter naming alone for comparison across models.

4 However, see other arguments that lower thresholds of specificity (e.g., 0.50) are acceptable for classification in the context of response-to-intervention instructional approaches (e.g., Catts et al. 2009).
